# Analysis of clinical data of different endometrial pathological types in perimenopausal women with abnormal uterine bleeding

**DOI:** 10.3389/fonc.2024.1370681

**Published:** 2024-02-29

**Authors:** Li Wang, Shimin Quan, E. Bai, Xiaofeng Yang

**Affiliations:** Department of Gynecology and Obstetrics, The First Affiliated Hospital of Xi’an Jiaotong University, Xi’an, Shaanxi, China

**Keywords:** perimenopause, abnormal uterine bleeding, endometrial cancer, history risk factors, ultrasonic imaging characteristics, ovarian cyst

## Abstract

**Objective:**

Early detection and diagnosis are important for improving the therapeutic effect and quality of life in patients with endometrial cancer (EC). This study aimed to analyze the clinical data of different endometrial pathological types in perimenopausal women with abnormal uterine bleeding (AUB) in order to provide evidence for the prevention and early diagnosis of EC.

**Methods:**

A total of 462 perimenopausal women with AUB were enrolled in this prospective observational study. Endometrial biopsy was performed in patients with suspected endometrial lesions. According to the pathological examination results, the patients were divided into endometrial polyp group (EP) (n = 71), endometrial hyperplasia without atypia group (EH) (n = 59), atypical endometrial hyperplasia (AEH) (n = 36), and EC group (n = 27). The history risk factors and ultrasonic imaging characteristics of endometrium among the four groups were compared.

**Results:**

Twenty-seven women were diagnosed with EC (5.84%). The prevalence rate of AEH and EC in the group of 51- to 55-year-old women was significantly higher than that in the groups of 40- to 45-year-old women and of 46- to 50-year-old women (*P <* 0.05). The age, body mass index, and history of diabetes gradually increased with the development of endometrial pathological types. In addition, the correlation index of endometrial blood flow increased gradually, and the proportion of uneven endometrial echo, unclear endometrial–myometrial junction (EMJ), and ovarian cyst also increased gradually. However, no statistically significant difference was found when comparing endometrial thickness (ET) and endometrial volume (EV) among endometrial pathological groups (*P >* 0.05). The ET, EV, endometrial vascularization index, endometrial flow index, and vascularization flow index in the ovarian cyst group were significantly higher (*P <* 0.05), and the proportion of uneven endometrium echo and unclear EMJ were significantly higher compared with that in the non-ovarian cyst group (*P <* 0.05).

**Conclusions:**

The most common cause of perimenopausal women with AUB was benign endometrial lesions. However, women aged 51–55 years old with endometrial high risk factors and ovarian cyst should be alert to AEH and EC. Endometrial biopsy needs to be performed to determine endometrial malignancy in necessity.

## Introduction

In recent years, women with risk factors for endometrial lesions have gradually risen, and the incidence rate of endometrial cancer (EC) has also increased (1). According to the latest cancer statistics reported in the United States in 2020, EC has ranked the fourth of newly diagnosed cases of female malignant tumor (2). In many economically developed areas of China, EC has become the most common malignant tumor of female reproductive system, and the incidence is increasing year by year, such as Beijing, Shanghai, and Guangzhou (3). Studies have reported that most patients with EC are diagnosed at the early stage (80% of stage I patients), and the 5-year survival rate could reach 95%. However, with regional spread or distant metastasis, 5-year survival was significantly reduced to 68% and 17%, respectively (4). Therefore, early detection, diagnosis, and treatment are essential for improving the therapeutic effect and quality of life in patients with EC.

It has been reported that the high incidence of EC is perimenopausal and postmenopausal women (5). Perimenopause is the physiological transition period that every woman has to go through. During this period, most women are in the state of rare ovulation or anovulation due to the decline of ovarian function. Therefore, they are prone to menstrual disorders and even abnormal uterine bleeding (AUB) (6). Although the endometrial lesions of most patients are benign, there are still some patients who progress from benign to EC because of continuous action of estrogen without progesterone to transform endometrium, especially in those who accompany risk factors of endometrium such as recurrent vaginal bleeding, early menarche, overweight or obesity, diabetes, hypertension, history of polycystic ovary syndrome, insulin resistance, estrogen-secreting tumors, infertility, estrogen therapy without progesterone supplement, Lynch syndrome, and use of Tamoxifen (7). This study aimed to analyze the etiology of perimenopausal women with AUB and to discuss the relationship between different pathological types of endometrium and the history of risk factors, as well as the characteristics of endometrial ultrasound imaging, in order to provide evidence for the prevention and early diagnosis of EC.

## Materials and methods

### Study design

We conducted a prospective observational study on perimenopausal women with AUB in the outpatient clinic of gynecology and women’s health care. All participants gave a written informed consent on the basis of procedures granted by the Ethics Committee of The First Affiliated Hospital of Medical College of Xi’an Jiaotong University (No.XJTU1AF2018LSK-218). This study has been registered on Clinical Trials.gov (ChiCTR03289468).

### Participants

A total of 462 perimenopausal women with AUB in The First Affiliated Hospital of Xi’an Jiaotong University from January 2018 to December 2020 were recruited for this prospective observational study. Women had a median age of 47.39 years with an age range of 40–55 years. In addition, the duration of AUB in all participants had median time of 14.49 days with a range of 3–30 days. The inclusion criteria were as follows: (a) Perimenopausal women aged ≥ 40 years with abnormal frequency of menstrual cycle, abnormal duration of menstruation, abnormal menstrual bleeding, or abnormal regularity of menstrual cycle; (b) bleeding from the uterine cavity; and (c) women with complete clinical data. Patients were excluded if they had bleeding not from the uterine cavity, such as genital tract infection or cervical lesions. Patients were also excluded if they had incomplete medical records. Questionnaire was used to record the basic information of all subjects, including chief complaint, history of present disease, age, body mass index (BMI), waist-to-hip ratio (WHR), menstrual history, reproductive history, family history, medical treatment history, menopausal hormone therapy (MHT) history, diabetes, and hypertension.

### Outcome measures

**Figure 1:**
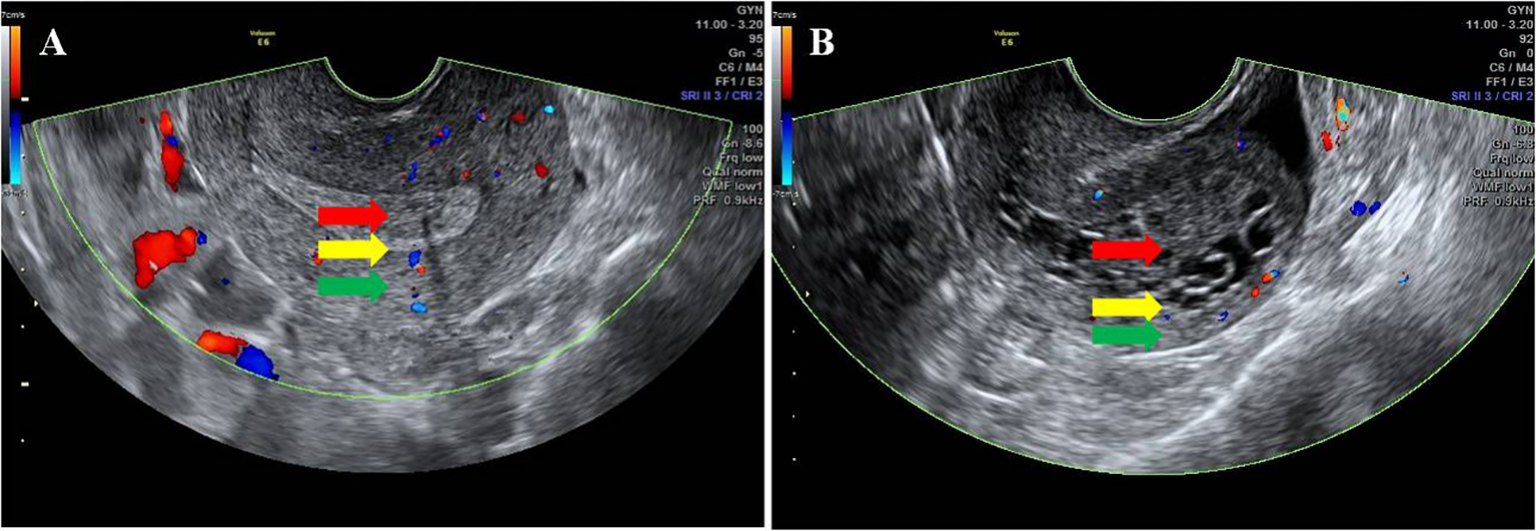
Ultrasonic characteristics of the EMJ. The red arrow indicates the endometrium, the green arrow indicates the myometrium, and the yellow arrow indicates the EMJ. **(A)** Clear EMJ. **(B)** Unclear EMJ.

The transvaginal ultrasound scanning (TVS) was performed using Voluson E8 (GE Medical Systems, USA). The maximum endometrial thickness (ET) on both sides of the midline was measured by transvaginal two-dimensional (2-D) ultrasonography. The endometrial echogenicity was observed, and the boundary between endometrium and myometriums was identified. The endometrial–myometrial junction (EMJ) is regarded as the boundary between endometrium and myometrium, which is thought to play an important role in both physiological and pathophysiological processes within the uterus. It has been suggested that a careful ultrasound examination of the EMJ can yield clinically useful information in a number of different pathologies (8). In this study, the EMJ was observed and evaluated in the longitudinal plane of the uterus, and the result was divided into clear (two borders were clearly visible) and unclear (one border was not clearly visible or two borders were not clearly visible) (**Figure 1**).

Afterward, the endometrial volume (EV), vascularization index (VI), flow index (FI), and vascularization flow index (VFI) were detected using three-dimensional (3-D) power Doppler ultrasound. The EV, VI, FI, and VFI were calculated automatically by Virtual Organ Computer-Aided Analysis (VOCAL) software (9). VI manifests the number of vessels in the endometrium. FI estimates the average intensity of flow. VFI manifests a combination of vascularity and blood flow. All ultrasonic examinations were performed in our hospital. The observer was blinded to any previous findings or diagnoses prior to the ultrasonic examination. In addition, all ultrasonic scans were performed by one operator to avoid inter-observer variation. The ultrasonic parameters were measured three times, and the average value was used for the final statistical analysis.

All ultrasonic examinations were performed immediately upon the patient’s visit. Because most perimenopausal women presented with ovulation dysfunction, the ultrasonic evaluation of most women were in the endometrial proliferative stage of the menstrual cycle in this study. Dilatation and curettage (D&C) and hysteroscopy were conducted in patients who were suspected endometrial lesions examined by transvaginal 2-D ultrasound or 3-D power Doppler ultrasound. Pathological examination was carried out by the pathology department of our hospital. The etiology of AUB was determined according to the medical history, physical examination and assay-assisted examination, and the PALM-COEIN system published in International Federation of Gynecology and Obstetrics 2011 was used to classify the etiology of AUB (10). All perimenopausal women with AUB were divided into three groups depending on the age of women: 40- to 45-year-old group (n = 139), 46- to 50-year-old group (n = 202), and 51- to 55-year-old group (n = 121). The endometrial pathological examination results and prevalence rate of endometrial lesions among the three groups were compared. Furthermore, patients with endometrial lesions were divided into four groups according to pathological examination results: endometrial polyps (EP) (n = 71), endometrial hyperplasia without atypia (EH) (n = 145), atypical endometrial hyperplasia (AEH) (n = 36), and EC (n = 27).

**Figure 2:**
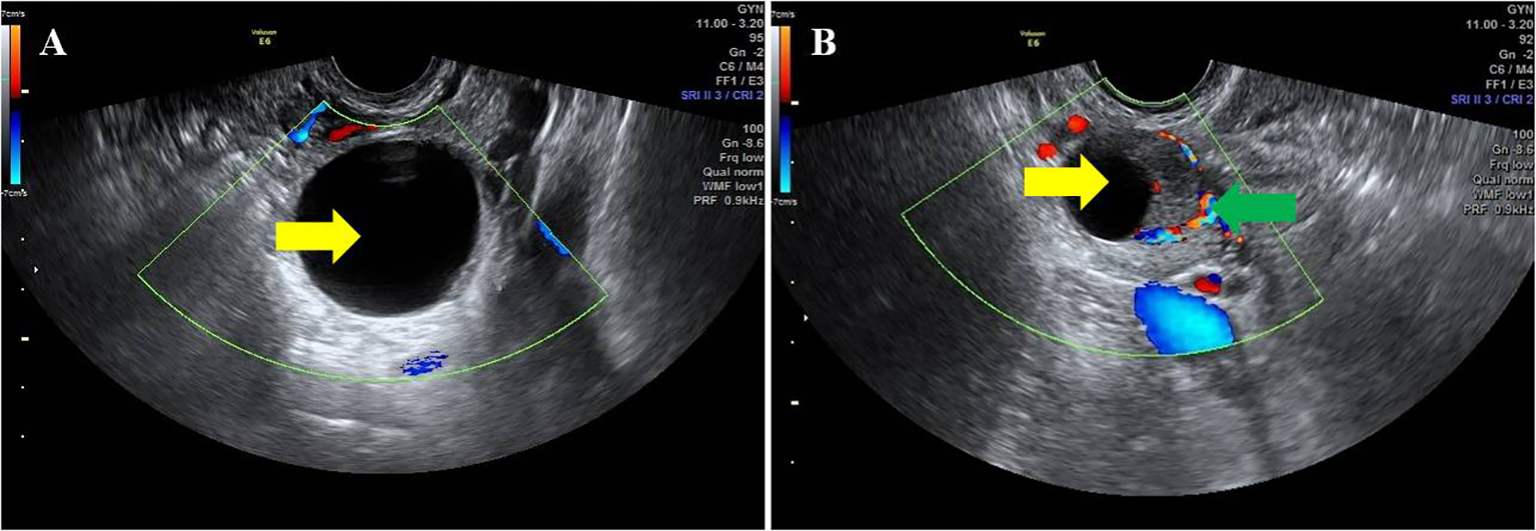
Ultrasonic characteristics of different ovarian cysts. The yellow arrow indicates ovarian cysts. The green arrow indicates the hemicycle blood flow around the corpus luteum. **(A)** Follicular cyst. **(B)** Corpus luteal cyst.

Two hundred and seventy-nine patients with different endometrial pathology in this study were divided into two groups according to whether ovarian cyst was detected by ultrasound. A total of 31 perimenopausal patients with AUB were found to have ovarian cysts by TVS. Follicular cyst and corpus luteal cyst are considered to be common functional or physiologic cysts, and both can occur during the menstrual cycle. Therefore, it is important to identify their ultrasonic characteristics. Follicular cyst was diagnosed as follows: regular shape, clear boundary, smooth cyst wall, thin-walled, internal fluid echo, and no blood flow signal. Corpus luteal cysts can appear complex or simple and thick-walled or can contain internal debris. Therefore, corpus luteal cyst was diagnosed as follows: regular shape, clear boundary, smooth or rough cyst wall, thick-walled, internal fluid echo or mixed echo, and, especially, hemicycle or annular blood flow signal (11) (**Figure 2**). Among the women enrolled in this study, the average diameters of cysts were 2–4 cm. The ultrasound imaging features of these cysts were regular shape, clear boundary, smooth cyst wall, internal fluid echo, and no blood flow signal. The relationship between ovarian cyst and endometrial ultrasound imaging was observed.

### Statistical analyses

Statistical analyses were performed using the SPSS version 18.0. The Kolmogorov–Smirnov test was used to check the normal distribution prior to statistical tests. The continuous variables were presented as mean ± standard deviation (SD) and analyzed by variance analysis or Student’s t-test. Differences in dichotomous outcomes were presented as number and percentage (%), which were analyzed by chi-square test or Fisher’s exact probability test. A value of *P <* 0.05 was considered statistically significant.

## Results

### Etiology of perimenopausal patients with AUB

**Figure 3:**
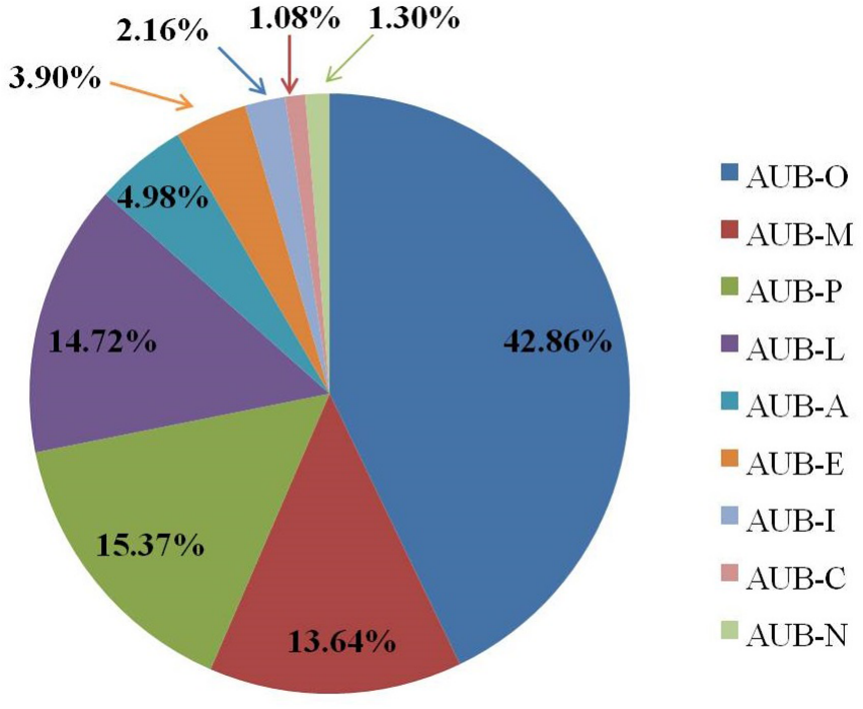
Etiological distribution of perimenopausal patients with AUB.

A total of 462 perimenopausal patients with AUB were included in this study. **Figure 3** shows the etiology of all participants; among them, benign endometrial lesions were the main cause. Meanwhile, AUB-O accounted for the highest proportion (198 cases, 42.86%), followed by AUB-P (71 cases, 15.37%), AUB-L (68 cases, 14.72%), and AUB-M (63 cases, 13.64%), AUB-A (23 cases, 4.98%), AUB-E (18 cases, 3.90%), AUB-I (10 cases, 2.16%), AUB-N (six cases, 1.30%), AUB-C (five cases, 1.08%). Twenty-seven perimenopausal patients with AUB were diagnosed with EC (5.84%).

### Endometrial pathological examination in women with different age groups

**Figure 4:**
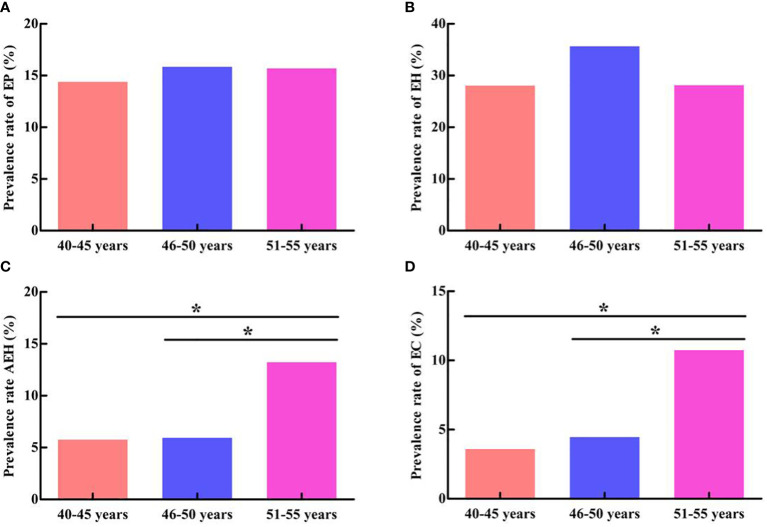
Comparison of endometrial pathological results among different age groups. **(A)** Comparison of EP prevalence rate. **(B)** Comparison of EH prevalence rate. **(C)** Comparison of AEH prevalence rate. **(D)** Comparison of EC prevalence rate. *P < 0.05.

**Table 1** presents the number and prevalence rates of endometrial pathological results among different age groups. Furthermore, the comparison of the prevalence rates of endometrial pathological results among different age groups is displayed in **Figure 4**. The data showed that the prevalence rate of AEH and EC in the 51- to 55-year-old group was significantly higher than that in the 40- to 45-year-old group and the 46- to 50-year-old group (*χ^2 ^= *4.306, *P* = 0.038; *χ^2 ^= *5.127, *P* = 0.024; *χ^2 ^= *5.069, *P* = 0.024; *χ^2 ^= *4.714, *P* = 0.030). However, no significant difference was found when comparing the prevalence rate of AEH and EC between the 46- to 50-year-old group and the 40- 45-year-old group (*χ^2 ^= *0.005, *P* = 0.943; *χ^2 ^= *0.154, *P* = 0.695). In addition, no significant difference was found in the prevalence rate of EP and EH among the three groups (*χ^2 ^= *0.148, *P* = 0.929; *χ^2 ^= *3.022, *P* = 0.221).

**Table 1:** The number and prevalence rates of endometrial pathological results among different age groups.

Pathological type	40- to 45-year-old group (n = 139)	46- to 50-year-old group (n = 202)	51- to 55-year-old group (n = 121)
EP	20 (14.39)	32 (15.84)	19 (15.70)
EH	39 (28.06)	72 (35.64)	34 (28.10)
AEH	8 (5.76)	12 (5.94)	16 (13.22)
EC	5 (3.60)	9 (4.46)	13 (10.74)

Data are presented as number (percentage).

### General data of patients with different types of endometrial pathology

The data showed that the most patients had benign endometrial lesions, including EP (n = 71, 15.37%), EH (n = 145, 31.39%), and AEH (n = 36, 7.79%), whereas the incidence rate of EC was 5.84% (n = 27). The data in **Table 2** suggest that age, BMI, and history of diabetes gradually increased with the development of endometrial pathological types. Patients with EC had maximum mean age and BMI, as well as the highest prevalence rate of diabetes (*P <* 0.05). However, no significant difference was found when comparing the other general data among the four groups (*P >* 0.05).

**Table 2:** Comparison of general data among patients with different pathological types of endometrium.

Characteristics	EP group (n = 71)	EH group (n = 145)	AEH group (n = 36)	EC group (n = 27)	*F/χ^2^*	*P-*value ^a^
Menarche age (years)	13.45 ± 7.65	12.45 ± 8.02	13.12 ± 9.03	12.10 ± 7.43	0.785	0.213
Age (years)	46.76 ± 9.02	47.09 ± 8.79	51.23 ± 9.05	54.39 ± 10.22	3.576	0.025
Gravidity (number)	4.23 ± 0.98	3.75 ± 0.85	3.90 ± 0.77	3.46 ± 0.73	0.897	0.142
Parity (number)	2.04 ± 0.54	1.98 ± 0.61	2.12 ± 0.85	1.67 ± 0.59	0.907	0.113
BMI (kg/m^2^)	22.35 ± 8.05	21.90 ± 7.98	22.09 ± 8.32	24.15 ± 8.56	2.687	0.046
WHR	0.81 ± 0.19	0.82 ± 0.18	0.80 ± 0.19	0.84 ± 0.20	1.209	0.098
Diabetes					7.845	0.049
Yes	2 (2.82)	6 (4.14)	4 (11.11)	4 (14.81)		
No	69 (97.18)	139 (95.86)	32 (88.89)	23 (85.19)		
Hypertension					4.421	0.219
Yes	5 (7.04)	10 (6.70)	4 (11.11)	5 (18.52)		
No	66 (92.96)	135 (93.10)	32 (88.89)	22 (81.48)		
History of MHT					1.775	0.620
Yes	7 (9.86)	12 (8.28)	5 (13.89)	4 (14.81)		
No	64 (90.14)	133 (91.72)	31 (86.11)	23 (85.19)		

BMI, body mass index; WHR, waist-to-hip ratio; MHT, menopausal hormone therapy.

Data are presented as mean ± SD or number (percentage).

a Variance analysis, chi-square test, or Fisher’s exact probability test.

### Ultrasonic characteristics of endometrium in patients with different endometrial pathological types

**Table 3** indicates the ultrasonic characteristics of endometrium in patients with different endometrial pathological types. There were statistically significant differences in endometrial VI, FI, VFI, endometrial echo, EMJ, and ovarian cyst among patients with different endometrial pathological types (*P <* 0.05), whereas no statistically significant difference was found when comparing endometrial ET and EV (*P >* 0.05). The data indicate that, with the development of endometrial pathological types, the correlation index of endometrial blood flow increased gradually. Moreover, the proportion of uneven endometrial echo, unclear EMJ, and ovarian cyst also increased gradually.

**Table 3:** Comparison of ultrasonic characteristics among patients with different pathological types of endometrium.

Parameters	EP group (n = 71)	EH group (n = 145)	AEH group (n = 36)	EC group (n = 27)	*F/χ^2^*	*P-*value ^a^
ET (mm)	6.34 ± 0.98	7.12 ± 1.03	9.07 ± 1.45	10.28 ± 1.96	1.807	0.063
EV (cm^3^)	4.15 ± 1.05	4.87 ± 1.12	5.34 ± 1.36	5.79 ± 1.54	1.906	0.059
VI	0.41 ± 0.09	0.45 ± 0.10	0.63 ± 0.15	0.75 ± 0.12	3.090	0.038
FI	22.25 ± 5.78	23.76 ± 6.10	26.37 ± 7.21	28.56 ± 8.32	2.787	0.042
VFI	0.10± ± 0.05	0.11 ± 0.07	0.17 ± 0.09	0.21 ± 0.13	3.239	0.034
Endometrial echo					67.452	0.000
Uniform	64 (90.14)	61 (42.07)	14 (38.89)	3 (11.11)		
Non-uniform	7 (9.86)	84 (57.93)	22 (61.11)	24 (88.89)		
EMJ					15.501	0.001
Clear	69 (97.18)	142 (97.93)	33 (91.67)	22 (81.48)		
Unclear	2 (2.82)	3 (2.07)	3 (8.33)	5 (18.52)		
Ovarian cyst					17.962	0.000
Yes	4 (5.63)	12 (8.28)	6 (16.67)	9 (33.33)		
No	67 (94.37)	133 (91.72)	30 (83.33)	18 (66.67)		

ET, endometrial thickness; EV, endometrial volume; VI, vascularization index; FI, flow index; VFI, vascularization flow index; EMJ, endometrial-myometrial junction.

Data are presented as mean ± SD or number (percentage).

a Variance analysis, chi-square test, or Fisher’s exact probability test.

### Relationship between ovarian cyst and endometrial ultrasound imaging

The patients with different endometrial pathological results in this study were divided into two groups according to whether ovarian cyst was detected by ultrasound. The data in **Table 4** show that the ET, EV, VI, FI, and VFI of endometrium in ovarian cyst group were significantly higher compared with patients in the non-ovarian cyst group (*P <* 0.05). Furthermore, the proportion of uneven endometrium echo and unclear EMJ was significantly higher than that the in non-ovarian cyst group (*P <* 0.05). In addition, 15 perimenopausal women with ovarian cysts obtained pathological results due to surgical treatment for AEH or EC, which were diagnosed as follicular cyst.

**Table 4:** Relationship between ovarian cyst and endometrial ultrasound imaging.

Parameters	Non-ovarian cyst group (n = 248)	Ovarian cyst group (n = 31)	*t/χ^2^*	*P*-value ^a^
ET (mm)	8.03 ± 1.12	12.36 ± 2.65	3.324	0.034
EV (cm^3^)	3.89 ± 1.06	5.56 ± 1.47	2.908	0.041
VI	0.35 ± 0.12	0.65 ± 0.17	3.124	0.039
FI	19.05 ± 6.23	25.09 ± 7.32	3.078	0.040
VFI	0.08 ± .04	0.16 ± 0.07	3.549	0.029
Endometrial echo			11.378	0.001
Uniform	158 (63.71)	10 (32.26)		
Non-uniform	90 (36.29)	21 (67.74)		
EMJ			4.241	0.039
Clear	76 (30.65)	4 (12.90)		
Unclear	172 (69.35)	27 (87.10)		

ET, endometrial thickness; EV, endometrial volume; VI, vascularization index; FI, flow index; VFI, vascularization flow index; EMJ, endometrial-myometrial junction.

Values are presented as mean ± SD or number (percentage).

a t-test, chi-square test, or Fisher’s exact probability test.

## Discussion

In recent years, with the change of living and eating habits and the increase of metabolic diseases, women with high risk factors of EC is also increasing (12). Therefore, EC is currently the most common malignancy of the female genital tract in developed countries around the world, as well as in some developed cities in China (13, 14). Although EC still occurs more commonly in older women, for whom the mortality rate is increasing, it also is being diagnosed in young women (15). Epidemiological data show that the incidence and mortality of EC are increasing globally, with the highest incidence in North America and Eastern Europe and the highest mortality in Melanesia and the Caribbean (16). At present, there are still controversies about the screening objects for EC around the world. Because of the lack of large sample data to confirm the feasibility of EC screening, most scholars and institutions point out that EC and precancerous lesion screening is more likely to be carried out in women with AUB and high risk factors of EC. The American Cancer Society guidelines in 2017 suggested that women with high risk factors of EC should be concerned if they have AUB and also should be screened for EC (17). Similarly, the guidelines published by the Japan Oncology Society recommend that women aged over 50 years should be screened for EC if they have AUB (18).

AUB often occurs in perimenopausal women, and it is one of the primary symptoms of EC (19). The etiology of endometrial lesions in 462 perimenopausal women with AUB was analyzed in this study. The data showed that the most patients had benign endometrial lesions, including EP (15.37%), EH (31.39%), and AEH (7.79%), whereas the incidence of EC was 5.84%. Meanwhile, AUB-O accounted for the highest proportion (42.86%), followed by AUB-P (15.37%) and AUB-L (14.72%). The ovarian function gradually declines in perimenopausal period, and most women are ovulation dysfunction. Therefore, endometrial hyperplasia occurs due to the continuous action of estrogen without progesterone antagonism (20). Nevertheless, the pathological change of endometrium from EH to AEH and then to EC is a continuous process, during which some women may reverse themselves or through progesterone therapy. Hence, endometrial lesions in most perimenopausal women with AUB are benign. In addition, this study compared the prevalence rate of benign and malignant endometrial lesions in different age groups. The data showed that the prevalence rate of AEH and EC in 51- to 55-year-old group was significantly higher than that in 40- to 45-year-old group and 46- to 50-year-old group. However, no significant difference was found when comparing the prevalence rate of AEH and EC between the 46- to 50-year-old group and the 40- to 45-year-old group. Furthermore, there no significant difference was found in the prevalence rate of EP and EH among the three groups, which are similar to the results reported in most literatures. The international cancer data survey showed that the age of high incidence of EC was 50–59 years old, the incidence rate of EC increased with the increase of age, and the relative risk of EC increased two to three times (21).

Increasing age, obesity, diabetes, early menarche, late menopause, recurrent episodes of postmenopausal bleeding, and unopposed use of exogenous estrogens without progesterone have been reported to be associated with an increased risk of EC (7, 12). The data in this study suggest that age, BMI, and history of diabetes gradually increased with the development of endometrial pathological types. Patients with EC had maximum mean age and BMI, as well as the highest prevalence rate of diabetes. Hence, lifestyle modifications such as proper diet and exercise can reduce the risk of developing obesity and metabolic diseases, as well as decrease the incidence of EC (22). However, there was no significant difference when comparing menarche age, gravidity number, parity number, WHR, prevalence rate of hypertension, and history of MHT among the four groups. This difference may be explained by differences in race, place of residence, life-style, sample size, and use of screening methods. Perimenopausal women are characterized by decline of ovarian function, ovulation dysfunction, and increased metabolic disorders (glucose and lipid metabolism) (20). Hence, the endometrial hyperplasia in perimenopausal women occurs directly or indirectly under continuous stimulation of estrogen, which can progress to endometrial atypical hyperplasia or even malignancy, especially in perimenopausal women with AUB. Endometrial hyperplasia is a disordered proliferation of endometrial glands. It results from the unopposed estrogenic stimulation of the endometrial tissue with a relative deficiency of the counterbalancing effects of progesterone (23). Endometrial hyperplasia, if not treated, has the propensity to develop into EC. Therefore, endometrial assessment and screening are necessary in these women in order to detect the possibility of any underlying malignancy.

There are several methods to identify the endometrial lesions, such as TVS, hysteroscopy, endometrial biopsy, and endometrial cytology (24). TVS is known as the first-line method in the evaluation of endometrium. However, 2-D Doppler ultrasound suffers from low specificity, due to its low positive predictive value and high false-positive rate. Hysteroscopy and D&C are second-line methods that have been considered as the gold standard in the diagnosis of endometrial pathology (25). Studies have shown that hysteroscopy play a major role in the diagnosis of endometrial malignancy (26). The occurrence of EC is the cancerous changes of endometrial epithelial cells under the action of multiple factors, which accompanied by abnormal proliferation of blood vessels. The change from EH to EC is accompanied with neovascularization and angiogenesis, which can be detected by 3-D power Doppler ultrasound (27). Very small vessels or vessels with irregular course can be accurately evaluated by power Doppler. Therefore, 3-D power Doppler angiography has been considered recently as new ultrasonic diagnostic tools of tumor. This technology can detect the EV, endometrial blood vessels, and blood flow by VOCAL software, so the following blood flow parameters can be calculated automatically: VI, FI, and VFI, which are not influenced by subjective factors (28). Therefore, 3-D power Doppler ultrasound is superior to 2-D color Doppler imaging for detecting low-velocity flows and visualizing small vessels. Mercé et al. reported that that EV and endometrial flow parameters detected by 3-D power Doppler were significantly higher in EC compared with EH (29). In addition, studies have reported that 3-D power Doppler parameters were more useful than ET for differentiating between EH and EC, and the blood flow in malignant tumor could predict the spread of EC (30). Similarly, the data in this study revealed that there were statistically significant differences in endometrial VI, FI, VFI, endometrial echo, EMJ, and ovarian cyst among patients with different endometrial pathological types, whereas no statistically significant differences were found when comparing endometrial ET and EV. The data indicated that, with the development of endometrial pathological types, the correlation index of endometrial blood flow increased gradually, and the proportion of uneven endometrial echo, unclear EMJ, and ovarian cyst also increased gradually.

In this study, our findings revealed that the ET, EV, VI, FI, and VFI in perimenopausal women with ovarian cyst were significantly higher than those without ovarian cyst. The most ovarian cyst in perimenopausal women was considered to be follicular cyst, which was concerned with ovulation dysfunction. The ultrasound imaging features of these cysts were regular shape, clear boundary, smooth cyst wall, thin-walled, internal fluid echo, and no blood flow signal. Although, these cysts need to be followed up and do not require medical treatment. However, their cyst wall is composed of granulosa cells, follicular membrane cells, or degenerative fibrous tissue. This kind of cyst can secrete estrogen autonomously and may cause precocious puberty in childhood, irregular menstruation in childbearing age and perimenopause, and abnormal vaginal bleeding after menopause (31). Therefore, continuous production of estrogen by ovarian cyst can stimulate endometrial hyperplasia continuously, which increases the risk of endometrial lesions. In this study, 15 perimenopausal women with ovarian cysts obtained pathological results due to surgical treatment for AEH or EC, which were diagnosed as follicular cyst.

Our study has some limitations. First, this is a single-center study. Second, the endometrial pathology results were not obtained in perimenopausal women with normal endometrium checked by ultrasound. Third, the examinations of ovarian cysts in women with normal endometrium or benign endometrial lesions were limited to ultrasound imaging evaluation, and their pathological data were not obtained in this study. In fact, these women with ovarian cysts are currently being followed up to monitor the endometrium and ovarian cysts. Finally, the results of this study remain to be studied in multi-center large sample research and further confirmed.

## Conclusions

This study displayed that, although the most common cause of perimenopausal women with AUB was benign endometrial lesions, women aged 51–55 years old with endometrial high risk factors or ovarian cyst should be alert to AEH and EC. Therefore, endometrial pathological examinations of these women need to be performed to determine endometrial malignancy in necessity.

## Data availability statement

The original contributions presented in the study are included in the article/supplementary material. Further inquiries can be directed to the corresponding author.

## Ethics statement

The studies involving humans were approved by The Ethics Committee of The First Affiliated Hospital of Medical College of Xi’an Jiaotong University (No.XJTU1AF2018LSK-218). The studies were conducted in accordance with the local legislation and institutional requirements. The participants provided their written informed consent to participate in this study.

## Author contributions

LW: Conceptualization, Writing – original draft, Writing – review & editing. SQ: Investigation, Writing – original draft. EB: Data curation, Formal Analysis, Writing – review & editing. XY: Writing – review & editing.
